# Postpartum pelvic floor muscle training, levator ani avulsion and levator hiatus area: a randomized trial

**DOI:** 10.1007/s00192-022-05406-z

**Published:** 2022-11-23

**Authors:** Gunvor Hilde, Jette Stær-Jensen, Franziska Siafarikas, Marie Ellström Engh, Kari Bø

**Affiliations:** 1 Department of Rehabilitation Science and Health Technology, Faculty of Health Sciences, OsloMet – Oslo Metropolitan University, Pilestredet, P.O. Box 4, St. Olavs plass, NO-0130 Oslo, Norway; 2 Department of Obstetrics and Gynecology, Akershus University Hospital, Lørenskog, Norway; 3 Division Akershus University Hospital, Faculty of Medicine, University of Oslo, Oslo, Norway; 4 Department of Sports Medicine, Norwegian School of Sport Sciences, Oslo, Norway

**Keywords:** Levator ani muscle avulsion, Levator hiatus area, Postpartum pelvic floor muscle training, Physical therapy, Vaginal delivery

## Abstract

**Introduction and hypothesis:**

Vaginal delivery may lead to tearing of the levator ani (LA) muscle from its bony insertions (complete LA avulsion) and increased levator hiatus (LH) area, both risk factors for pelvic floor dysfunctions. Early active rehabilitation is standard treatment after musculo-skeletal injury. We hypothesized that pelvic floor muscle training (PFMT) early postpartum would reduce the presence of LA avulsions and reduce LH area.

**Methods:**

We carried out a planned secondary analysis from a randomized controlled study. Primiparous women (*n*=175) giving birth vaginally were included 6 weeks postpartum, stratified on complete LA avulsion, and thereafter randomized to PFMT or control. The training participants (*n*=87) attended a supervised PFMT class once a week and performed home-based PFMT daily for 16 weeks. The control participants (*n*=88) received no intervention. Presence of complete LA avulsion, LH area at rest, maximal contraction, and maximal Valsalva maneuver were assessed by transperineal ultrasound. Between-group comparisons were analyzed by analysis of covariance for continuous data, and relative risk (RR) for categorical data.

**Results:**

Six months postpartum, the number of women who had complete LA avulsion was reduced from 27 to 14 within the PFMT group (44% reduction) and from 28 to 17 within the control group (39% reduction). The between-group difference was not significant, RR 0.85 (95% CI 0.53 to 1.37). Further, no significant between-group differences were found for LH area at rest, during contraction, or Valsalva.

**Conclusions:**

Supervised PFMT class combined with home exercise early postpartum did not reduce the presence of complete LA avulsion or LH area more than natural remission.

## Introduction

**Fig. 1:**
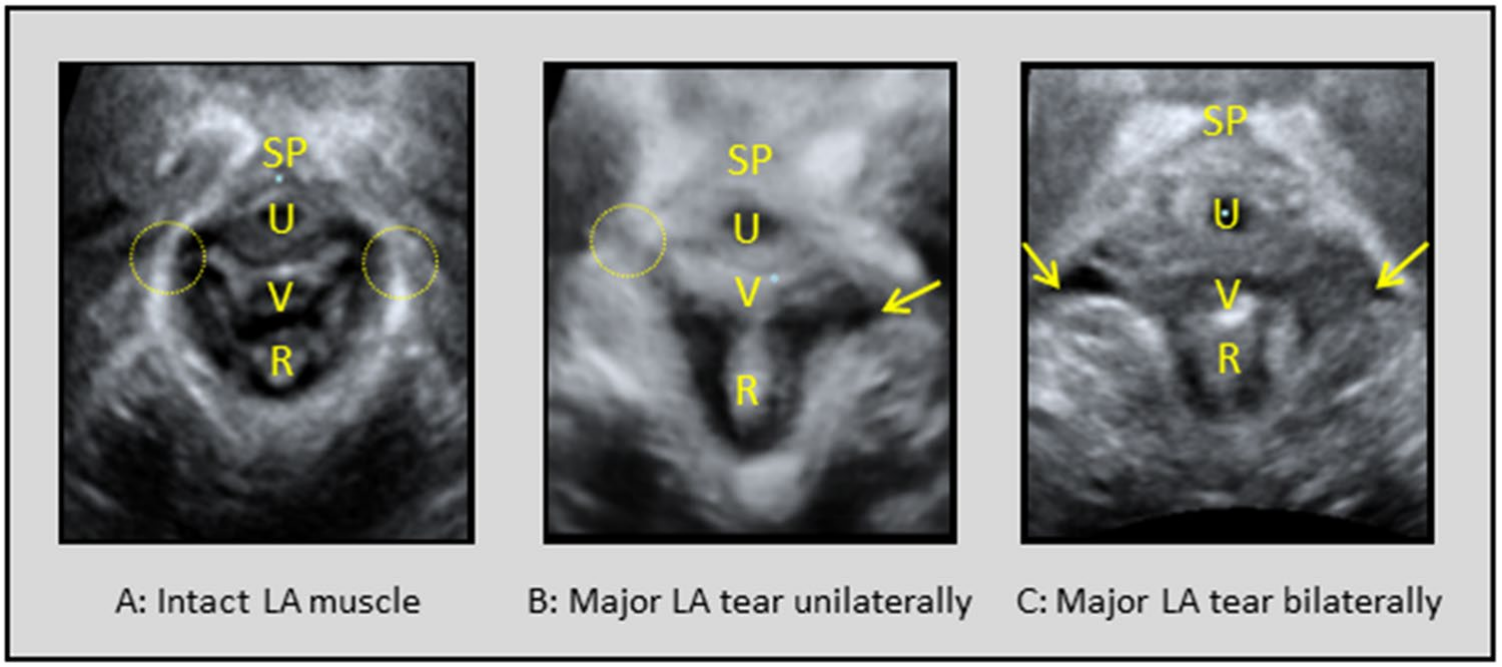
Tomographic ultrasound imaging of the levator ani (*LA*) muscle after vaginal delivery in the axial plane of minimal hiatal dimensions in render mode [19]. Normal muscle insertion of the LA muscle is marked by the *dotted circle*. Abnormal LA muscle insertion is marked by an arrow. Image **A** shows an intact LA muscle, **B** shows a major LA tear unilaterally, and **C** shows a major LA tear bilaterally. *SP* symphysis pubis, *U* urethra, *V* vagina, *R* rectum

The most medial muscle portion of the levator ani (LA) muscle borders the levator hiatus (LH), an opening in the pelvic floor allowing the passage of the urethra, rectum, and the vagina [1]. During vaginal delivery the medial muscle fibers of the LA muscle might be stretched up to three times their resting length as the fetal head is crowning [2]. This significant degree of distension may result in a complete avulsion of the LA muscle [3], seen as a complete visible muscle detachment from the pubic bone either unilaterally or bilaterally (Fig. 1). According to imaging studies using transperineal ultrasound or magnetic resonance imaging a complete LA muscle avulsion (unilaterally or bilaterally) may be present in 13–36% of the primiparous women giving birth vaginally [4]. LA avulsion might be accompanied by enlargement of the LH [5, 6], decreased pelvic floor muscle (PFM) strength [7–9] and less ability to decrease the LH area during PFM contraction [10]. This major muscle injury has been linked to pelvic floor dysfunction later in life and pelvic organ prolapse in particular [4, 11]. In recent publications from a longitudinal study, in which participating women were recruited 5–10 years after their first childbirth and thereafter assessed for pelvic floor dysfunction yearly for up to 9 years [11, 12], the strong association between complete LA avulsion and pelvic organ prolapse could to a large extent be explained by a larger LH area and decreased PFM strength.

Early active rehabilitation is standard treatment after musculo-skeletal injury within sports physical therapy, and training is believed to be important in speeding up tissue healing (repair and remodeling) [13]. This approach is supported by experimental studies showing that early mobilization and training after muscle injury may facilitate capillary ingrowths, improve orientation of the regenerating myofibrils, and improve tensile properties [14]. The managing of soft-tissue injuries within sport might give new directions in understanding how soft tissue injury caused by childbirth can recover [15]. So far there is scant knowledge on to what extent postpartum PFM training (PFMT) would reduce the presence of complete LA avulsion and reduce the LH area.

The main aim of the present study was to investigate whether PFMT early after vaginal delivery enhances tissue healing, seen as a reduction in number of complete LA avulsions detected via ultrasound (primary outcome), and a reduction of the absolute value of the LH area measured at rest, and during maximal PFM contraction and Valsalva (secondary outcomes). An added aim was to explore whether women with complete LA avulsion have a larger LH area during a PFM contraction than women without such major tears at baseline and after the intervention ended.

## Materials and methods

This article is a planned secondary analysis from a parent study, an assessor-blinded randomized controlled trial (RCT), conducted to assess the effect of postpartum PFMT on urinary incontinence [16]. The study counted 175 primiparous women and was conducted at Akershus University Hospital, Norway, from February 2010 to May 2012. Data were collected at 6 weeks and 6 months postpartum. In this secondary analysis we studied the effect of PFMT with regard to changes in the presence of complete LA avulsion and changes in the absolute LH area.

Power calculation and sample size determination was done for the primary outcome of the parent study, urinary incontinence [16]. Thus, specific power calculations for reduction in number of complete LA avulsion or LA area were not performed.

### Participants

As described in the parent study [16], the inclusion criteria were primiparous women giving birth vaginally to a singleton infant after more than 32 weeks of gestation and being able to speak and understand Scandinavian languages. Exclusion criteria were prior abortion or stillbirth after 16 weeks of gestation, serious illness to the mother or child, or perineal tears grade 3C and higher where more than 50% of the external anal sphincter is torn [17]. Women presenting with severe perineal tearing are routinely referred to physical therapy for PFM training at our hospital and could therefore not be allocated to the control arm of the study.

Demographic data were collected through electronic questionnaires and obstetrical data were collected from the women’s medical birth records. Prior to the study start all participants had received a written leaflet containing information on postpartum PFMT from the postnatal ward. When included (6 weeks postpartum), ahead of randomization, all participants were instructed by one of two trained physical therapists (GH, KG) in how to perform a correct PFM contraction. Correct contraction was defined as a cranioventral shift of the LA muscle (inward lift and squeeze around the urethra, vagina, and rectum), and was verified by observation and vaginal palpation [18]. Whether they were able to contract correctly was coded Yes/No.

### Ultrasound examination

After the initial learning session on how to correctly contract the PFM, the participants were examined with three- and four-dimensional transperineal ultrasound by one of two trained investigators (JSJ, FS). A GE Voluson E8 system (GE Medical Systems, Zipf, Austria), with a 4– to 8-MHz curved-array volume transducer (RAB4-8l/obstetric) was used. After voiding, the participant was positioned in a standardized supine lithotomy position, a position used throughout the whole study when acquiring ultrasound volumes. Ultrasound images were acquired at rest, during maximal PFM contraction and maximal Valsalva maneuver, using a previously described methodology [6]. Three maximal PFM contractions and three maximal Valsalva maneuvers were recorded. Care was taken to avoid co-contraction of the PFM during the Valsalva maneuver. The same ultrasound examination took place after the intervention ended (6 months postpartum).

#### Complete LA avulsion

As suggested by Dietz et al. [19], tomographic imaging of the axial plane of maximal PFM contraction was used to assess complete LA avulsion and diagnosed when an abnormal insertion of the muscle toward the pubic bone were present in three central slices, either unilaterally or bilaterally. Data on complete LA avulsion (primary outcome) were collapsed into two categories (LA avulsion/no LA avulsion). For women unable to contract the LA muscle, tomographic imaging of the rest volumes was used to assess muscle integrity. Two trained assessors (JSJ, FS) performed the ultrasound assessment on LA muscle tearing. They were blinded to the women’s obstetric history, prior examinations, and ultrasound assessments. The inter-rater agreement between these two investigators was good to excellent (Cohen’s kappa ranging from 0.63 to 0.91) [20].

#### LH area measurements

**Fig. 2:**
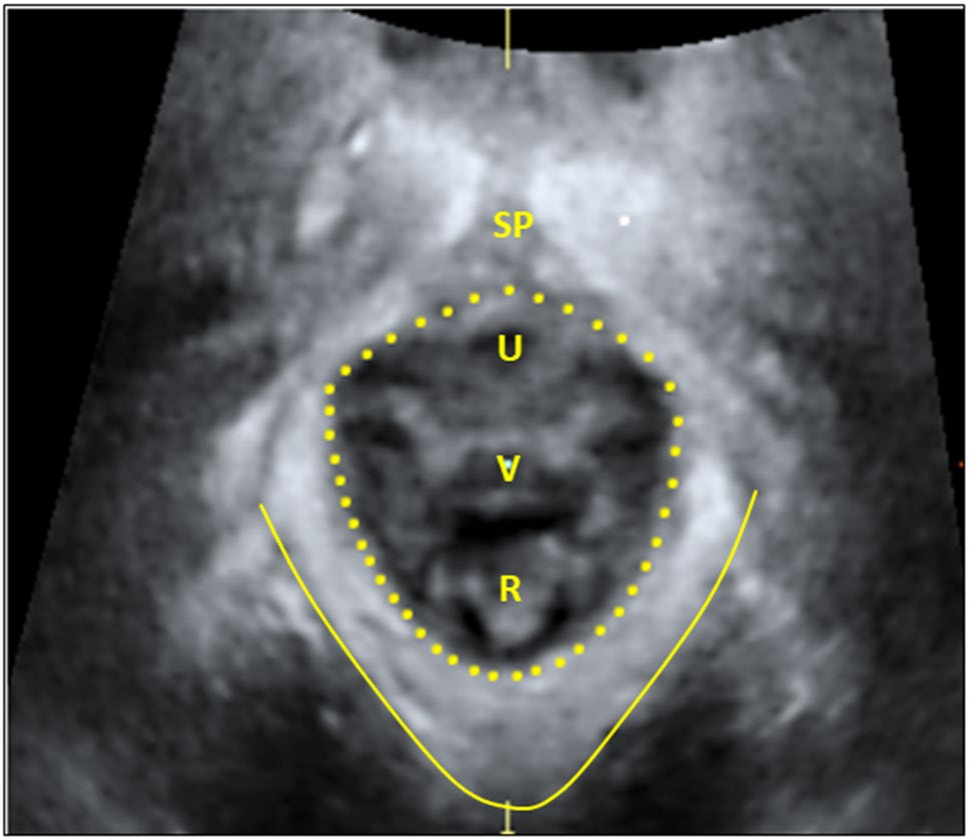
The levator ani muscle seen between the *solid and dotted line* in the axial plane of minimal hiatal dimensions in render mode [19]. The muscle inserts on the os pubis bilaterally of the symphysis pubis (*SP*) and forms a “U-shaped” sling around the urethra (*U*), vagina (*V*), and rectum (*R*), and hence border the levator hiatus area (seen within the *dotted line*)

In this article we report on the absolute values of the LH area. Render mode around the plane of minimal hiatal dimensions was used when measuring the LH area [19]. For contraction, the volume with the best contraction, defined as the one with the shortest anteroposterior diameter from the posteroinferior margin of the symphysis pubis to the LA muscle in the midsagittal plane was chosen for analysis [21]. For the Valsalva maneuver, the volume with the largest anteroposterior diameter in the midsagittal plane was chosen for analysis [21]. Rest position was defined as the caudal-most position of the LA muscle before a PFM contraction. The LH area (Fig. 2) was measured as the area bordered by the LA muscle, the symphysis pubis, and the inferior ramus pubis [22]. Although two trained investigators assessed tearing of the LA muscle, four trained investigators (JSJ, FS, GH, KG) measured the LH area, with an intraclass correlation coefficient ≥ 0.80 [21]. The same investigator measured the individual participant’s scan at both 6 weeks and 6 months postpartum in a blinded manner.

### Randomization

As described in the parent study, the participants were stratified on complete LA avulsion 6 weeks postpartum, and thereafter randomized into one of two groups (training or control) in blocks of 10 [16]. The computer-generated randomization sequence was concealed by opaque envelopes. The allocation of participants was administered outside the clinical room by a project midwife. The outcome assessors were kept blinded for group allocation throughout the study.

### Intervention

As outlined in the parent study [16], the training participants attended an intervention period of 16 weeks, consisting of a supervised PFMT class once a week led by an experienced physical therapist. The exercise class protocol is described in detail by Mørkved and Bø [23]. Additionally, the training group was prescribed to perform daily PFMT at home throughout the intervention period (three sets of 8–12 PFM contractions close to maximum). Training participants recorded their adherence at home in a training diary, whereas the physical therapist recorded group session adherence. Beyond the customary leaflet and the thorough initial instruction and assessment of correct PFM contraction ahead of randomization, the control group received no further intervention. Control participants were not discouraged from doing PFMT.

### Statistical analysis

Statistical analyses were performed using SPSS version 27 (SPSS, Chicago, IL, USA). Within-group comparisons were analyzed by paired Student’s *t* test for continuous data, and by McNemar’s test for categorical data. Between-group comparisons (training versus control) for continuous data were assessed by analysis of covariance (ANCOVA) taking into account variability of the of LH area at baseline, and also other variables showing imbalance at baseline if considered clinically relevant. Independent-sample Student’s *t* test was used for comparisons between groups (training versus control) at baseline and when comparing women with LA avulsion versus women with no LA avulsion (secondary aim). Between-group comparisons (training versus control) for categorical data were analyzed by Chi-squared test and relative risk (RR) ratio. *p* Values < 0.05 were considered significant. Intention to treat was the principal analysis. Missing values on continuous data (LH area) were imputed by using the participant’s baseline value plus added mean change observed in the corresponding control group. For major LA avulsion coded as “yes” or “no,” the approach of “last observation carried forward” was applied by using baseline data on LA avulsion. A per protocol analysis was also carried out, in which drop-outs, training participants with an exercise adherence ≤ 80%, and participants with a new pregnancy at the clinical visit 6 months after delivery were excluded.

There were no important changes to methods after trial commencement.

### Institutional review board

The study was approved by the Regional Medical Ethics Committee (REC South East Norway 2009/289a) and the Data Protect Officer at Akershus University Hospital, Lørenskog, Norway (2799004) and registered at ClinicalTrials.gov (NCT01069484). All subjects gave written informed consent before entering the study.

## Results

**Fig. 3:**
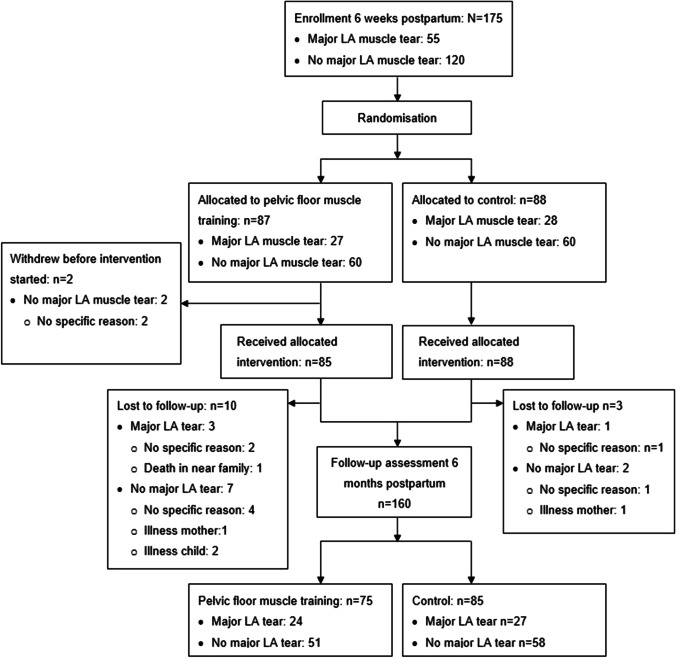
Flowchart of participants through each stage of the randomized trial. This flowchart has previously been published [16]

A total of 175 primiparous women were included 6 weeks postpartum. The stratum diagnosed with complete LA avulsion included 55 women and the stratum without such major tears included 120 women. Number of participants randomly allocated to PFMT and to control and further flow throughout the trial is shown in Fig. 3. Per stratified randomization, 27 and 28 participants with complete LA avulsion were allocated to the treatment and control groups respectively. Seven of the 175 women (4%) were not able to perform a correct PFM contraction at baseline (6 weeks postpartum); 4 were allocated to the training arm (3 of them had complete LA avulsion), and 3 to the control arm (one of them had complete LA avulsion). Twelve women in the training arm and 3 in the control arm were lost to follow-up. No harm or adverse effect from PFMT was reported.

Baseline characteristics of the study participants are presented in Table 1. A statistically significantly higher number of women in the control group had a higher education level than women in the training group, and their infants had higher birth weight. Other baseline characteristics were not significantly different between the groups.

**Table 1:** Baseline characteristics and delivery data of the primiparous women included

Characteristics	Total sample (*n*=175)	PFM training group (*n*=87)	Control group (*n*=88)	*p* value PFM vs control group
Demographics				
Age (years)	29.8 (SD 4.1)	29.5 (SD 4.3)	30.1 (SD 4.0)	0.376
Body mass index (kg/m^2^) 6 weeks	25.7 (SD 4.0)	26.0 (SD 4.1)	25.3 (SD 3.9)	0.262
Education				
College or university	143 (81.7%)	64 (73.6%)	79 (89.8%)	0.010
Primary school/, high school, other	32 (18.3%)	23 (26.4%)	9 (10.2%)	
Civil status				
Married or cohabitant	166 (94.9%)	80 (92.0%)	86 (97.7%)	0.099
Single	9 (5.1%)	7 (8.0%)	2 (2.3%)	
Delivery data				
Instrumental assisted delivery	35 (20%)	20 (23.0%)	15 (17.0%)	0.427
Length of second stage (min)^a^	68.8 (SD 46.3)	66.8 (SD 45.2)	70.8 (SD 47.5)	0.576
Infant birth weight (g)	3,462.5 (SD 454.2)	3,543.7 (SD 482.3)	3,382.3 (SD 411.7)	0.018
Infant head circumference (cm)^b^	34.8 (SD 1.6)	34.8 (SD 1.6)	34.9 (SD 1.5)	0.632
Unable to contract	7 (4%)	4 (2.3%)	3 (1.7%)	0.720
Complete LA avulsion^c^	55 (31.4%)	27 (31.0%)	28 (31.8%)	1.000

Continuous variables given as means (± standard deviation, SD), categorical variables as numbers (percentages, %)

Between-group comparisons were analyzed by independent sample Student’s *t* test (continuous data), and by Chi-squared test (categorical data). Some of these data have been previously published [16]

*PFM* pelvic floor muscle, *LA* levator ani

^a^Total *n*=172; missing data on 3 women; 1 from the training group; 2 from the control group

^b^Total *n*=174; missing data on 1 infant from the training group

^c^Complete LA avulsion: major tearing of the LA muscle seen as a complete visible muscle detachment from the pubic bone, either unilaterally or bilaterally [19]

For generalizability, the total population of primiparous women (*n*=2,621) scheduled for delivery at Akershus University Hospital during our inclusion period had a mean age of 28.4 years, 92.7% were married or cohabitant, and 50.8% had higher education (college or university) [16].

### Adherence

Recordings of PFMT adherence (class sessions and home training) showed that 96% of the training group participants reached an adherence level of 80%. Control participants were asked retrospectively about PFMT at the post-intervention test, and 16.5% reported having performed PFM training ≥ 3 times per week [16].

### Complete LA avulsion

No new cases of complete LA avulsion were detected 6 months postpartum. From baseline (mean 6.1 weeks after delivery, SD 0.9) to post-intervention (mean 6.1 months after delivery, SD 0.8) McNemar’s test showed that the number of women with complete LA avulsion was significantly reduced from 27 to 15 within the training group (44% reduction, *p*=0.002) and from 28 to 17 within the control group (39% reduction, *p*=0.001). The relative risk analysis (RR) 6 months postpartum in the stratum of women with complete LA avulsion (*n*=55) showed no significant difference in the presence of LA avulsion between the training group (*n*=27) and the control group (*n*=28), RR = 0.85 (95% CI 0.53 to 1.37).

### LH area measurements

Change from 6 weeks to 6 months postpartum within groups for LH area at rest, during maximal contraction and maximal Valsalva maneuver is shown in Table 2. Overall, there was a significant reduction in the LH area (absolute measures) from 6 weeks to 6 months within both groups, except for change at rest within both training and control in the complete LA avulsion stratum and for change during maximal Valsalva within training in the complete LA avulsion stratum (Table 2).

**Table 2:** The levator hiatus area at rest, during maximal pelvic floor muscle contraction, during and maximal Valsalva maneuver in primiparous women with and without a major tear of the levator ani muscle. The women participated in a controlled study on postpartum pelvic floor muscle (PFM) training running from 6 weeks (baseline) to 6 months postpartum. The participants were stratified on levator ani (LA) muscle avulsion and thereafter randomized to PFM training or control. The intention to treat analyses are shown

	LA avulsion stratum (*n*=55)				No LA avulsion stratum (*n*=120)			
	PFM training (*n*=27)	Control (*n*=28)	Corrected mean difference PFM training vs control (95% CI)*	*p* value	PFM training (*n*=60)	Control (*n*=60)	Corrected mean difference PFM training vs control (95% CI)*	*p* value
Levator hiatus area, rest (cm^2^)								
6 weeks postpartum	13.94 (2.93)	14.24 (3.53)	−0.35 (−2.17 to 1.47)	0.700	13.45 (2.73)	14.59 (2.89)	−1.12 (−2.29 to −0.16)	0.025
6 months postpartum	13.25 (2.41)	13.37 (3.19)	−0.14 (−1.27 to 0.99)	0.806	12.58 (2.45)	13.13 (2.57)	−0.15 (−0.55 to 0.85)	0.671
Change 6 weeks to 6 months	↓0.68 (−0.28 to 1.65)	↓0.87 (−0.09 to 1.83)			↓0.87 (0.39 to 1.35)	↓1.46 (0.89 to 2.04)		
Levator hiatus area, contraction (cm^2^)								
6 weeks postpartum	13.28 (2.67)	13.43 (3.35)	−0.24 (−1.93 to 1.45)	0.776	11.58 (2.28)	12.01 (2.31)	−0.45 (−1.33 to 0.43)	0.442
6 months postpartum	11.30 (2.06)	11.81 (3.16)	−0.55 (−1.55 to 0.45)	0.277	10.11 (2.23)	10.44 (1.81)	−0.07 (−0.52 to 0.38)	0.756
Change 6 weeks to 6 months	↓1.98 (1.20 to 2.76)	↓1.62 (0.75 to 2.49)			↓1.47 (1.14 to 1.79)	↓1.57 (1.21 to 1.93)		
Levator hiatus area, Valsalva (cm^2^)								
6 weeks postpartum	22.92 (7.34)	24.99 (6.59)	−1.98 (−5.84 to 1.88)	0.308	21.97 (6.33)^a^	22.47 (6.35)	−0.94 (−3.32 to 1.44)	0.435
6 months postpartum	21.65 (6.48)	21.50 (7.32)	1.44 (−1.57 to 4.45)	0.341	18.68 (5.35)	19.87 (6.07)	−0.73 (−2.22 to 0.76)	0.335
Change 6 weeks to 6 months	↓1.27 (−1.16 to 3.70)	↓3.49 (1.35 to 5.62)			↓3.30 (2.00 to 4.59)	↓2.60 (1.66 to 3.55)		

Levator ani (*LA*) avulsion indicates major tearing of the LA muscle seen as a complete visible muscle detachment from the pubic bone either unilaterally or bilaterally [19]

*PFM* pelvic floor muscle

Paired Student’s *t* test was used for within-group comparisons, values presented at 6 weeks and 6 months postpartum are means (standard deviations), change from 6 weeks to 6 months postpartum at mean difference (95% CI), ↓ means reduction

*ANCOVA was used for between-group comparisons with the following adjustments: at 6 weeks postpartum controlling for infant birth weight and education level; at 6 months controlling for baseline value of the LH area, infant birth weight, and education level. Values presented are estimated corrected means, also called marginal means (95% CI)

^a^One image volume missing at 6 weeks postpartum. Volume could not be analyzed because of low image quality

When performing the ANCOVA analyses we controlled for the participant’s education level and the infant’s birthweight in addition to the LH area at baseline. This was done because of an imbalance at baseline. No between-group differences (training versus control) were found, either in the complete LA avulsion stratum or in the stratum without complete avulsion (Table 2).

The per-protocol analysis produced similar results as the intention-to-treat analysis both for the presence of complete LA avulsion and for LH area measurements (data not shown).

### LH area in women with and without complete LA avulsion

Within both the training arm and the control arm of the RCT a significantly larger LH area during maximal PFM contraction was shown among women with complete LA avulsion, both at baseline and post-intervention (Table 3). Clinical assessment of ability to contract the PFM, showed that the 7 women who were not able to contract the PFM correctly 6 weeks postpartum had the same inability to contract the PFM 6 months postpartum.

**Table 3:** Differences in the levator hiatus area when comparing primiparous women with and without major tear of the levator ani (*LA*) muscle. Data from the training and control arms of a randomized trial on postpartum pelvic floor muscle training running from 6 weeks (baseline) to 6 months postpartum. The intention-to-treat analyses are shown

	Training arm (*n*=87)				Control arm (*n*=88)			
	LA avulsion (*n*=27)	No LA avulsion (*n*=60)	Mean difference avulsion vs no avulsion (95% CI)*	*p*value	LA avulsion (*n*=28)	No LA avulsion (*n*=60)	Mean difference avulsion vs no avulsion (95% CI)*	*p*value
Levator hiatus area, rest (cm^2^)								
6 weeks postpartum	13.94 (2.93)	13.45 (2.73)	0.49 (−0.80 to 1.78)	0.451	14.24 (3.53)	14.59 (2.89)	−0.35 (−1.76 to 1.06)	0.622
6 months postpartum	13.25 (2.41)	12.58 (2.45)	0.68 (−0.45 to 1.80)	0.233	13.37 (3.19)	13.13 (2.57)	0.24 (−1.02 to 1.50)	0.707
Change 6 weeks to 6 months	↓0.68 (−0.28 to 1.65)	↓0.87 (0.39 to 1.35)			↓0.87 (−0.09 to 1.83)	↓1.46 (0.89 to 2.04)		
Levator hiatus area, contraction (cm^2^)								
6 weeks postpartum	13.28 (2.67)	11.58 (2.28)	1.70 (0.59 to 2.81)	0.003	13.43 (3.35)	12.01 (2.31)	1.42 (0.01 to 2.83)	0.049
6 months postpartum	11.30 (2.06)	10.11 (2.23)	1.19 (0.19 to 2.19)	0.021	11.81 (3.16)	10.44 (1.81)	1.37 (0.07 to 2.67)	0.039
Change 6 weeks to 6 months	↓1.98 (1.20 to 2.76)	↓1.47 (1.14 to 1.79)			↓1.62 (0.75 to 2.49)	↓1.57 (1.21 to 1.93)		
Levator hiatus area, Valsalva (cm^2^)								
6 weeks postpartum	22.92 (7.34)	21.97 (6.33)^a^	0.95 (−2.13 to 4.02)	0.543	24.99 (6.59)	22.47 (6.35)	2.52 (−0.41 to 5.44)	0.091
6 months postpartum	21,65 (6.48)	18.81 (5.40)	2.84 (0.19 to 5.49)	0.036	21.50 (7.32)	19.87 (6.07)	1.63 (−1.32 to 4.59)	0.274
Change 6 weeks to 6 months	↓1.27 (−1,16 to 4.70)	↓3.30 (2.00 to 4.59)			↓3.49 (1.35 to 5.62)	↓2.60 (1.66 to 3.55)		

Levator ani (LA) avulsion indicates major tearing of the LA muscle seen as a complete visible muscle detachment from the pubic bone either unilaterally or bilaterally [19]

^a^One image volume missing at 6 weeks postpartum. Volume could not be analyzed because of low image quality

*Independent-sample Student’s *t* test was used for between-group comparisons (major tear versus no tear). Values presented within the groups at 6 weeks and 6 months postpartum are means (standard deviations), values between groups are mean differences (95% CI)

## Discussion

In this study we found no significant differences in the reduction of cases of complete LA avulsion when comparing participants allocated to the PFMT group versus participants allocated to the control group (*p*>0.05). However, the presence of complete LA avulsion decreased significantly within both groups. Six months postpartum, there was a 44% reduction of complete LA avulsion within the PFMT group and 39% reduction within the control group. Further, no significant between-group differences (training versus control) were found 6 months postpartum for LH area measures, not at rest, during maximal contraction or during maximal Valsalva maneuver. During PFM contraction, the strata of women with complete LA avulsion 6 weeks postpartum persisted in having a larger LH area 6 months postpartum than those of women without such muscle defects.

A search in PubMed revealed no former RCTs on postpartum PFMT with stratified analyses on LA avulsion. Further, RCTs on PFMT in which ultrasonography is used to assess outcome seem sparse. A former RCT [24] applying a similar PFMT intervention showed increased muscle thickness and decreased LH area at rest in favor of the training group. However, direct comparison is not possible, as Brækken et al. [24] included older women with pelvic organ prolapse, applied individual supervised PFMT, and did not perform stratified analysis on complete LA avulsion.

The PFMT intervention in the present study was based on strength training recommendations [25], but the results showed that our postpartum training program did not prove to reduce the LH area or the presence of LA avulsion more than natural remission. One possible explanation might be that it is hard to compete with a significant natural healing of the pelvic floor during the first postpartum year, especially from 6 weeks to 6 months postpartum [6, 26]. Previous findings from observational studies by our group and others show a 19–62% reduction of complete LA avulsion from 6 weeks to 1 year postpartum [27]. Another possible explanation might be that the initial thorough instruction on how to contract the PFM correctly resulted in an intervention effect for the control group too.

However, our results on complete LA avulsion within groups are in contrast to the longitudinal cohort study by Miller at al. [9], who report no reduction in the presence of major LA tears when following women from 7 weeks to 8 months postpartum. Comparison of results is somewhat limited owing to differences in study design. Miller at al. [9] included 68 women who had birth-related risk factors for LA muscle tears (long second stage, anal tears, and/or older maternal age). Further, they assessed LA tears by using magnetic resonance imaging (MRI) and categorized LA muscle tears somewhat differently than in our study. Transperineal ultrasound is a reliable tool and is established as the state-of-the-art methodology. However, diagnosing LA muscle tearing early after delivery might be challenging owing to tissue discrimination difficulty and poor image quality [27].

The applied supervised group PFMT program in our RCT may present a limitation, as findings in the parent study [16] showed no between-group differences in PFM function in terms of vaginal resting pressure, PFM strength, or endurance. Likewise, in this secondary analysis, we found no between-group differences in muscle morphology in terms of reduced LH area or presence of LA avulsion. Similar findings in the parent study and in this secondary analysis may not be surprising, as a moderate positive correlation between PFM strength and reduction of LH area was found by Bø et al. [28]. Specificity and overload are two fundamental principles that must be carefully addressed for effective PFMT [29, 30]. Even though the training intervention in our study followed known exercise science principles, we applied only one weekly supervised group session and other than that daily PFMT at home. It might be that our intervention was not monitored closely enough to reach the specificity and overload we were aiming at, especially for participating women with LA avulsion. It may be that postpartum women with such major muscle tears need a more continuous supervised individual instruction in order to achieve correct PFM contractions close to maximum, which is crucial in order to benefit from PFMT in terms of improving muscle strength [29, 30].

The LA muscle plays an important role in maintaining pelvic floor function; any alteration to the muscle may therefore impact pelvic floor function [1], and findings from a longitudinal study showing that the strong association between complete LA avulsion and pelvic organ prolapse could to a large extent be explained by a larger LH area and decreased PFM strength [11, 12]. Hence, there is a need for upcoming RCTs applying tailored treatment strategies, and a closer follow-up in terms of supervised individual instruction, as applied successfully in the study by Brækken et al. [24].

Further, we need to target women at risk for decreased ability to contract the PFM after vaginal childbirth [9]. During the acute healing phase for these women, we need better insight into whether certain activities should be scaled down or scaled up [15]. After the acute healing phase, these women should be offered interventions that can enable them to perform correct PFM contractions close to maximum to build on the potential capacity by non-injured muscle fibers to compensate for loss in muscle strength due to muscle tearing.

### Strength

As far as we have ascertained, this is the first RCT on postpartum PFMT on the morphological changes of the PFM after vaginal delivery in a study sample stratified on complete LA avulsion. In this study, ultrasound volumes have been analyzed by experienced examiners, and good to very good reliability has been shown. An added strength of the study was blinding of the outcome assessors, who also were blinded to delivery data. We used a PFMT program in which exercise science principles were followed [25].

As we wanted to investigate whether early active rehabilitation after vaginal delivery in terms of PFMT could speed up tissue healing [13, 14], we decided to include primiparous women only. This strengthens the study, as multiparous women may have been exposed to complete LA avulsion from a former childbirth and the intervention would not then be regarded as early active rehabilitation.

### Limitations

Compared with the total population scheduled for delivery at our hospital, the study sample had a higher level of education, which limits generalization of our results [16]. Furthermore, the inclusion criterion requiring Scandinavian language skills most likely caused a selection of participants, which also limits generalization of our results. It is estimated that one sixth of the 2,621 nulliparous pregnant women scheduled for delivery at Akershus University Hospital during the inclusion period were not eligible owing to the language criterion.

We aimed for 80 women with complete LA avulsion. However, time and resources prohibited us from reaching this goal. We managed to include only 55. This may represent a limitation with respect to statistical power [16]. Other than this, there were no changes to methods after trial commencement.

There was greater loss to follow-up in the training arm versus the control arm (12 vs 3), which may be an indicator of how realistic the tolerability to the PFMT regimen was for the patients. The participants brought their newborns to the supervised training sessions, and even though they were aware of this ahead of study start and thought it would be possible to attend the training intervention, it might have turned out otherwise. However, this is only speculation as we have no data on the reasons for withdrawal from the study.

## Conclusion

Supervised PFMT class once a week combined with home exercise after vaginal delivery did not reduce the presence of complete LA avulsion or LH area more than natural remission. Regardless of PFMT the presence of complete LA avulsion in primiparous women was significantly reduced from 6 weeks postpartum to 6 months. During PFM contraction, the strata of women with complete LA avulsion 6 weeks postpartum persisted, having a larger LH area 6 months postpartum than the strata of women without such muscle defects. Most women with complete LA avulsion are able to contract the PFM. Therefore, there is a potential capacity by non-injured muscle fibers to compensate for loss in muscle strength.
